# Co-Cultivation of Cross-Kingdom Microorganisms Effectively Triggers the Production of Tryptophol and Its Heterologous Expression in *E. coli*

**DOI:** 10.3390/microorganisms14040798

**Published:** 2026-04-01

**Authors:** Yue Li, Xiulei Xia, Jinwei Ren, Huarong Tan, Jine Li

**Affiliations:** 1 Beijing Institute of Dental Research, Beijing Stomatological Hospital, Capital Medical University, Beijing 100070, China; 2 State Key Laboratory of Microbial Diversity and Innovative Utilization, Institute of Microbiology, Chinese Academy of Sciences, Beijing 100101, China; 15668393609@163.com (X.X.); tanhr@im.ac.cn (H.T.); 3 State Key Laboratory of Mycology, Institute of Microbiology, Chinese Academy of Sciences, Beijing 100101, China; renjw@im.ac.cn

**Keywords:** microbial co-culture, silent gene clusters, natural products, tryptophol, heterologous expression

## Abstract

Genome sequencing has revealed that microorganisms have the potential to produce many more natural products than previously thought; the challenge is to establish efficient ways to “wake up” those “sleeping” biosynthetic pathways or genes, which are undoubtedly expressed in nature under specific conditions that are not normally reproduced in the laboratory. To activate these cryptic natural products, co-cultivation of cross-kingdom microorganisms between *Candida albicans* and *Streptomyces longshengensis* was performed in this study. A novel peak generated through co-culture was isolated and analyzed by a high-performance liquid chromatograph (HPLC), and its chemical structure was further determined by using mass spectrum (MS) and nuclear magnetic resonance (NMR) analyses. Bioassays of antimicrobial and antitumor activities were performed, and heterologous expression in *Escherichia coli* was attempted. The chemical structure was identified as tryptophol, and the bioassays revealed that tryptophol showed antitumor activity with IC_50_ values of 154.5, 144.3, 122.6, and 110.7 μg/mL against A549, MC38, HepG2, and MCF-7 cells, respectively. As a valuable compound, tryptophol was also heterologously expressed in *E. coli* C41 to address the drawbacks of chemical synthesis. These findings combine co-cultivation with genetic engineering for tryptophol biosynthesis, expanding its antitumor application and laying a foundation for its industrial and sustainable production.

## 1. Introduction

Fungi and bacteria, particularly actinomycetes, harbor vast reservoirs of natural products. As a crucial source of novel bioactive small-molecule drugs, they hold immense potential for development and application across clinical medicine, agriculture, animal husbandry, and other fields [1,2]. With the rapid development of third-generation whole genome sequencing technology, researchers have gained insight into the remarkable capacity of microbial communities to synthesize diverse secondary metabolites (SMs). Genome mining techniques have further uncovered numerous uncharacterized or non-expressed “silent” metabolic pathways in bacteria and fungi, whose products are referred to as silent SMs. As the discovery rate of new antibiotics declines and bacterial resistance rises, these silent metabolites have become invaluable resources for novel drug discovery [3,4,5]. Therefore, activating microbial secondary metabolic pathways is an essential strategy for exploring new SMs. In natural environments, microbial ecological relationships are complex, involving interactions such as coexistence, mutualism, symbiosis, antagonism, and other modes [6]. Standard laboratory cultures often fail to replicate this natural complexity, resulting in reduced discovery of SMs. Co-cultivation technology, which can trigger natural ecosystems and stimulate microorganisms to produce active metabolites, has thus emerged as a cutting-edge strategy for discovering new compounds [7]. For instance, two new papulacandin analogs, together with 12 known compounds, were identified from the co-culture of two endophytic fungi, *Pestalotiopsis* sp. J025 and *Nigrospora* sp. Wn-2-2 [8]. Diverse SMs were identified by gas chromatography coupled with mass spectrometry (GC-MS) through the co-cultivation of *Trichotomospora caesia* AC-1134 and *Streptoverticillium* sp. AC-1375; meanwhile, their antioxidant properties have been evaluated [9].

The activation mechanisms of cryptic gene clusters through co-cultivation technology are complex and can be categorized as follows: (1) Nutrient acquisition: small molecules that diffuse as precursors or substrates induce another strain to produce new metabolites; (2) Competition and signaling: using exogenous molecules as chemical stimulators, microbes produce antibiotics or signaling molecules to participate in competition; and (3) Physical contact: direct cell-to-cell interactions may activate the expression of silent gene clusters, although the specific mechanisms remain unclear [10,11]. The co-cultivation of inter-bacteria, inter-fungi, and cross-kingdom bacteria–fungi combinations are general modes of microbial communication: (1) Bacterial co-cultivation activated the biosynthesis of natural products—for example, when *Mycobacterium flavorhizae* and *Streptomyces cyanobacilli* were co-cultured under iron-limited conditions, competition for iron triggered the synthesis of actinorhodin in *Streptomyces cyanobacilli* [12]; (2) Fungal co-cultivation: co-cultivation of the basidiomycete *Phellinus orientoasiaticus* and *Xylodon flaviporus* successfully activated three new cyclohumulanoid sesquiterpenes along with five known analogs [13]; and (3) Fungal-bacterial co-cultivation presents greater complexity: co-cultivation of *Fusarium oxysporum* and *Streptomyces lisellans* on solid rice medium induced the production of four novel naphthoquinone dimer compounds (fusatricinones A-D) and a new pyranone derivative, dihydrolateropyrone [14].

In this study, we aim to select several bacteria and fungi as experimental materials to activate the expression of certain silent biosynthetic pathways, and the co-cultivation of *S. longshengensis* and *C. albicans* is performed. Tryptophol is not originally produced in the independent cultivation of the two strains, but it has been generated in the co-culture system of the two strains. Tryptophol and tryptophol analogs are closely linked to tryptophan metabolic pathways and exhibit diverse biological activities, including improving sleep, inhibiting pathogenic fungal growth, and inducing apoptosis. These properties not only make them highly promising for pharmaceutical and food applications, but also for serving as important drug intermediates for critical medicines such as the anti-inflammatory analgesic etodolac and anti-migraine drug sumatriptan [15]. Current tryptophol synthesis primarily relies on chemical methods, which are plagued by heavy pollution and high energy consumption. Therefore, microbial synthesis has been explored to solve these bottle-neck problems or improve yields. *Saccharomyces cerevisiae* was employed as a chassis cell to enhance the production of tryptophol, and the resulting yields were up to 478 mg/L [16]. Researchers significantly improved yields by modifying key genes in tryptophol biosynthetic pathways and optimizing fermentation conditions [17,18]. Compared with *Saccharomyces cerevisiae*, *Escherichia coli* shows prominent advantages such as lower costs, simpler cultivation, faster growth, and easier genetic manipulation, making it particularly valuable for biotechnological applications. Here, we utilize *E. coli* as a chassis cell to reprogram key biosynthetic genes involved in tryptophol biosynthesis. This study provides new insights into tryptophol activation and application and lays a foundation for the efficient synthesis of tryptophol in a microbial cell factory.

## 2. Materials and Methods

### 2.1. Experimental Strains and Cells

*Escherichia coli* JM109 was used for plasmid construction; *Escherichia coli* Arctic, Rossetta, BL21, and C41 were used as hosts for heterologous expression of tryptophol. *Streptomyces longshengensis* 4.1101, *C. albicans* 2.4159, *Staphylococcus aureus* 1.89, *Bacillus subtilis* 1.1630, *Pseudomonas aeruginosa* PAO1, and *Burkholderia cepacia* 1.1813 were used for co-cultivation. *Streptococcus pneumoniae* 010, *Pseudomonas aeruginosa* PA14, *Streptococcus pyogenes* #2, and *Bacillus subtilis* 1.1630 were used as indicator strains for analyzing the bioassays of antibacterial activities. *S. longshengensis* 4.1101, *C. albicans* 2.4159, *Staphylococcus aureus* 1.89, *Bacillus subtilis* 1.1630, and *Burkholderia cepacia* 1.1813 were purchased from CGMCC (China General Microbiological Culture Collection Center, Beijing, China). *Pseudomonas aeruginosa* PAO1 (NCBI Taxonomy ID 208964) and *Pseudomonas aeruginosa* PA14 (NCBI Taxonomy ID: 652611) were provided by Professor Lvyan Ma (Institute of Microbiology, Chinese Academy of Sciences, Beijing, China). *Streptococcus pneumoniae* 010 and *Streptococcus pyogenes* #2 were provided by Professor Zhoujie Xie (Tianjin University of Science and Technology, Tianjin, China). The *E. coli* Arctic Express strain was provided by Professor Yihua Chen (Institute of Microbiology, Chinese academy of sciences, Beijing, China). Human lung cancer cells (A549), mouse colorectal cancer cells (MC38), human liver cancer cells (HepG2), human breast cancer cells (MCF-7), and the human acute monocytic leukemia cells (THP-1) (BeNa Culture Collection, Beijing, China) were utilized for cytotoxicity assays of tryptophol. The plasmid pET28a:P_T7_-*aro9AC-aro10AC-adh1AC* was used for IPTG-inducible expression of tryptophol.

### 2.2. Strains Cultivation

Cultivation conditions for *S. longshengensis* and *C. albicans*: 30 μL of *C. albicans* was inoculated into 3.5 mL of liquid PDB (potato dextrose broth) medium (potato extract powder 5 g/L, glucose 15 g/L, tryptone 10 g/L, NaCl 5 g/L) in a glass tube and cultured overnight at 37 °C and 220 rpm. To enlarge cultivation, 200 μL of this culture was transferred to 10 mL of liquid PDB medium and incubated under the same conditions. The *S. longshengensis* strain was revived on MS agar plates (20 g/L mannitol, 20 g/L soybean meal, 1.5–2% (*w*/*w*) agar) until sporulation occurred, then co-cultured with *C. albicans* in an edge-contact manner. Other conditional pathogenic bacteria including *Staphylococcus aureus* 1.89, *Bacillus subtilis* 1.1630, *Pseudomonas aeruginosa* PAO1, and *Burkholderia cepacia* 1.1813 were cultivated in LB medium (tryptone 10 g/L, yeast extract 5 g/L, NaCl 10 g/L, pH 7.4). Co-culture conditions for these bacteria followed the above methods.

Co-cultivation conditions for product production: The MS solid medium was sterilized and poured into 90 mm × 15 mm petri dishes. We inoculated 20 μL of pre-prepared *C. albicans* seed suspension and 50 μL of *S. longshengensis* spore suspension on each half of the MS agar plate in an edge-contact manner. The plates were incubated at 28 °C for 6–8 days. The detector wavelengths of HPLC were set at 210 nm, 254 nm, 280 nm, and 310 nm.

Cultivation conditions for *E. coli* C41: The culture of *E. coli* C41/pET28a or *E. coli* C41/pET28a::P_T7_-*aro9AC*-*aro10AC*-*adh1A*C was inoculated with 1% of the volume into modified M9 medium in shake flask and incubated at 37 °C for 2–3 h until the OD_600_ reached 0.6–0.8, and then IPTG with a final concentration of 0.1–1 mM was added to induce protein expression. The preparation of modified M9 medium was as follows: yeast extract 5 g/L, glucose 20 g/L, disodium hydrogen phosphate 6.78 g/L, potassium dihydrogen phosphate 3 g/L, ammonium chloride 1 g/L, sodium chloride 0.5 g/L, magnesium chloride 0.12 g/L, calcium chloride 0.11 g/L, vitamin B1 10 mg/L, and 1000× trace metal solution at 1 mL/L composed of H_3_BO_3_ 2.86 g/L, MnCl_2_·4H_2_O 1.81 g/L, ZnSO_4_·7H_2_O 0.222 g/L, NaMoO_4_·2H_2_O 0.39 g/L, CuSO_4_·5H_2_O 0.079 g/L, and Co(NO_3_)_2_·6H_2_O 49.4 mg/L.

### 2.3. Isolation and Purification of Compound for Large-Scale Co-Cultivation

For obtaining crude extract from co-cultured agar plates, the co-cultured MS medium was incubated for 6–8 days, then cut into small pieces and collected in a jar. An equal volume of ethyl acetate was added, and ultrasonic extraction was performed for 1 h. The crude extract was concentrated using a rotary evaporator. This extraction process was repeated three times. The dried extract was dissolved in 2 mL of chromatographic-grade methanol and transferred to a clean 2 mL Eppendorf tube. After centrifugation at 13,000 rpm for 10 min and filtration through a 0.22 μm filter membrane, the compound was analyzed by HPLC (high-performance liquid chromatograph, Prominence LC-20A, Shimadzu, Kyoto, Japan) equipped with a ZORBAX SB-C18 reverse phase column (4.6 × 250 mm, 5 μm, Agilent, CA, USA). The flow rate was set at 1.0 mL/min using a linear gradient elution of acetonitrile and water, with the details being listed in Table 1. The detector wavelength was set at 280 nm.

To determine the chemical structure of the compound, a large amount of metabolite LSBN-280 should be first prepared from the large-scale co-culture fermentation medium. The method is described as follows.

For the initial isolation of compound, 30 L of MS agar was used for the co-culture of *S. longshengensis* and *C. albicans*. After 6–8 days, the MS solid medium was cut into small pieces and collected in several 3 L jars, and it was extracted with ethyl acetate to obtain crude extracts as described previously. The crude extracts were first passed through a vacuum chromatography column (40 mm diameter, 300 mm length, packed with 200–300 mesh silica gel). A linear gradient elution with 10–90% (*v*/*v*) acetonitrile aqueous solution was applied. The effluent was evaporated to dryness and re-dissolved in methanol, and then separated in the HPLC process, which was divided into two parts: semi-preparative column separation and analytical column separation. For semi-preparative column separation, the samples were processed using a HPLC semi-preparative column (ZORBAX SB-C18 reverse phase column, 5 μm, 9.4 × 250 mm, Agilent, CA, USA) through two rounds of separation. The flow rate was 3 mL/min at room temperature, with the injection volume adjusted to maximum capacity. The effluent was collected, evaporated to dryness, and re-dissolved in methanol. Detailed information on HPLC separation conditions is listed in Table 2.

For the third round of separation, the final purification conditions were optimized over 60 min using a C18 analytical column (ZORBAX SB-C18, 5 μm, 4.6 × 250 mm, Agilent, CA, USA) with 17% (*v*/*v*) acetonitrile. The flow rate was set as 1 mL/min.

### 2.4. Structural Identification of Compound by MS and NMR

MS analysis of LSBN-280 was performed on a Xevo G2-XS QToF mass spectrometer (Waters, Manchester, UK) in positive ion mode using an Acquity UPLC BEH C18 column (2.1 mm × 50 mm, 1.7 µm, Waters, Manchester, UK). The eluent consisted of acetonitrile and water containing 0.1% formic acid, applied in a variable linear gradient as detailed in Table 3. The resulting mass spectrum data were analyzed by Masslynx V 4.1 software.

NMR analysis: The purified compound was dissolved in deuterated DMSO and analyzed using NMR spectroscopy. The ^1^H and ^13^C NMR spectra were recorded on a Bruker Avance III 500 spectrometer (Bruker, Fällanden, Switzerland). The data were analyzed by MestReNova software (version topspin 3.5), and the results were consistent with those of tryptophol.

### 2.5. Antibacterial Bioassays of Tryptophol Against Several Bacterial Strains

To detect the biological activities of tryptophol against *Streptococcus pneumoniae* 010, *Pseudomonas aeruginosa* PA14, *Streptococcus pyogenes* #2, and *Bacillus subtilis* 1.1630, different concentrations of tryptophol dissolved in 50 µL of solvent (methanol:water = 1:1) were added into the holes (0.8 cm in diameter) of LB agar medium containing 1% (*v*/*v*) indicator strains, respectively. The maximum concentration of tryptophol was 1 mg/mL, and then it was serially diluted at a 1:1 ratio. The plates were incubated at 37 °C for 10–15 h, and the antibacterial activities were estimated by measuring the diameter of the inhibition zones.

### 2.6. Cell Counting Kit-8 (CCK-8) Cytotoxicity Assay

CCK-8 Assay: The cytotoxic effects of tryptophol on the A549, HepG2, MC38, MCF7, and THP-1 cell lines were evaluated using CCK-8 assays. A549 cells were cultured in DMEM (Dulbecco’s modified Eagle medium) supplemented with 10% FBS (fetal bovine serum) and 1% P/S (penicillin-streptomycin solution); HepG2 cells were cultured in MEM (minimum essential medium) supplemented with 10% FBS and 1% P/S; MC38 cells were cultured in DMEM + 10% FBS + 1% MEM NEAA (non-essential amino acids) + 1% sodium pyruvate + 1% GlutaMAX-1 + 1% HEPES + 1% P/S; MCF-7 cells were cultured in MEM supplemented with 10% FBS and 1% P/S; and THP-1 cells were cultured in RPMI-1640 with 10% FBS, 1% (2 mM) L-Glutamine, and 1% P/S. Cells were first revived and diluted to a concentration of 1 × 10^5^ cells/mL. Then, 100 μL of the cell suspension was added to each well of a 96-well plate and cultured in a 5% CO_2_ incubator at 37 °C for 24 h to allow for adherent growth. A tryptophol stock solution was prepared in DMSO at a concentration of 80 mg/mL and serially diluted 1:1 to obtain a range of tryptophol concentrations. Adherent cells were then treated with 100 μL of these tryptophol solutions, resulting in a final DMSO concentration of 0.5%. Depending on the cell growth rates, cultures were incubated for 48 or 72 h. Subsequently, CCK-8 reagent (containing WST-8) was added to each well and incubated for an additional 30 min to 1 h. Finally, the optical density at 450 nm (OD_450_) was measured using a microplate reader (PerkinElmer, Connecticut, USA) to generate a dose–response curve [19].

## 3. Results

### 3.1. Co-Cultivation of Cross-Kingdom S. longshengensis and C. albicans Leads to the Emergence of a New Compound Peak

**Figure 1:**
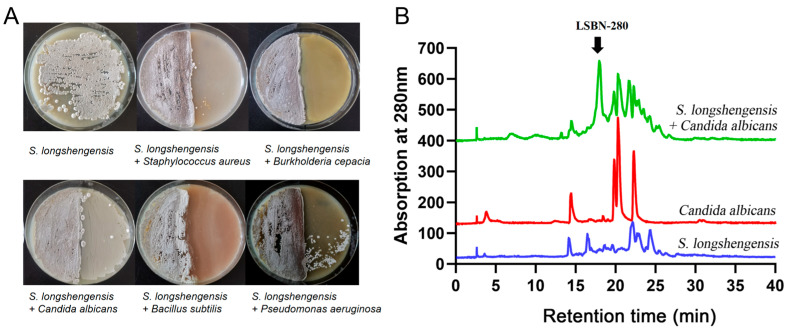
Co-cultivation of *S. longshengensis* 4.1101 and *C. albicans* 2.4159 led to the appearance of a new characteristic peak. (**A**) Co-cultivation of *S. longshengensis* and several pathogenic microorganisms on MS solid plates; (**B**) HPLC analysis of crude extracts from single and co-cultivated strains. Ultraviolet (UV) absorption at 280 nm is illustrated.

Since bacteria and fungi can produce numerous metabolites, we aimed to discover new compounds by co-culturing *S. longshengensis* with several conditional pathogenic microorganisms, including *Staphylococcus aureus* 1.89, *Burkholderia cepacia* 1.1813, *C. albicans* 2.4159, *Bacillus subtilis* 1.1630, and *Pseudomonas aeruginosa* PAO1. HPLC analysis revealed that the emergence of a new compound peak in the co-cultivation of *S. longshengensis* 4.1101 and *C. albicans* 2.4159 has attracted our attention, whereas the independently cultured *S. longshengensis*, *C. albicans*, and other co-cultured combinations showed no significant differences. Through repetition of the experiments (Figure S1), we determined that the distinct peak appearing at 18 min could be produced stably at the absorption of 280 nm, which was abbreviated as LSBN-280 (Figure 1).

### 3.2. Structural Identification of the Compound Produced Through the Co-Cultivation of S. longshengensis and C. albicans

**Figure 2:**
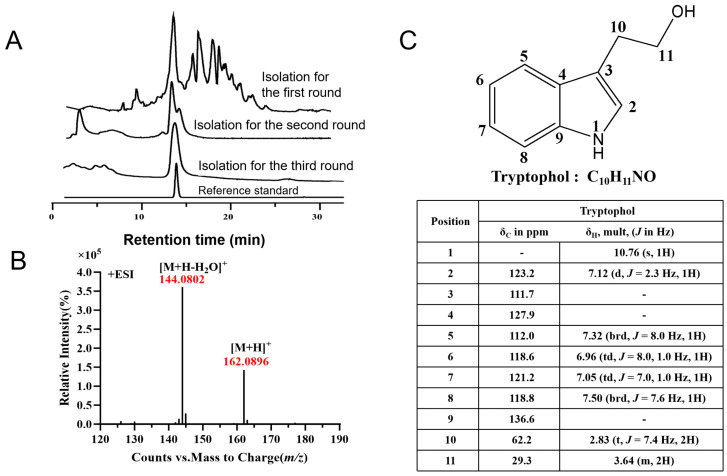
Isolation and structural identification of LSBN-280 from the co-cultivation of *S. longshengensis* and *C. albicans.* (**A**) Three-round separation and purification process of LSBN-280; (**B**) structural identification of LSBN-280 by HRESI-MS in positive ion mode; (**C**) ^1^H NMR and ^13^C NMR spectra data of LSBN-280.

To determine the structure of the compound, three batches of large-scale fermentation were conducted using approximately 30 L of MS solid medium over 6–8 days. The MS solid medium was cut into small pieces and placed in large glass beakers or shaking flasks, followed by extraction three times with ethyl acetate. The combined extracts were concentrated using a rotary evaporator, dissolved in methanol, and then purified through HPLC using semi-preparative and analytical columns over three rounds of separation (Figure 2A). To ensure that the purity of target compound LSBN-280 exceeded 95% and fit the requirements for NMR detection, 2 mg of high-purity LSBN-280 was characterized by high-resolution electrospray ionization mass spectrometry (HRESI-MS) and NMR spectroscopy. MS analysis of the purified LSBN-280 was performed on a Xevo G2-XS QToF mass spectrometer in positive ion mode. The [M + H]^+^ of LSBN-280 was m/z 162.0896 by HRESI-MS (Tryptophol, C_10_H_11_NO) (Figure 2B). The ^1^H and ^13^C NMR data of LSBN-280 matched those of tryptophol (Figure 2C) [20]. Taken together, LSBN-280 was identified as tryptophol. Subsequently, we established standard curves (Figure S2) and calculated that the yield of tryptophol in the co-culture system reached 11.96 mg/L, which remained relatively low.

### 3.3. Determination of the Biological Activities of Tryptophol

**Figure 3:**
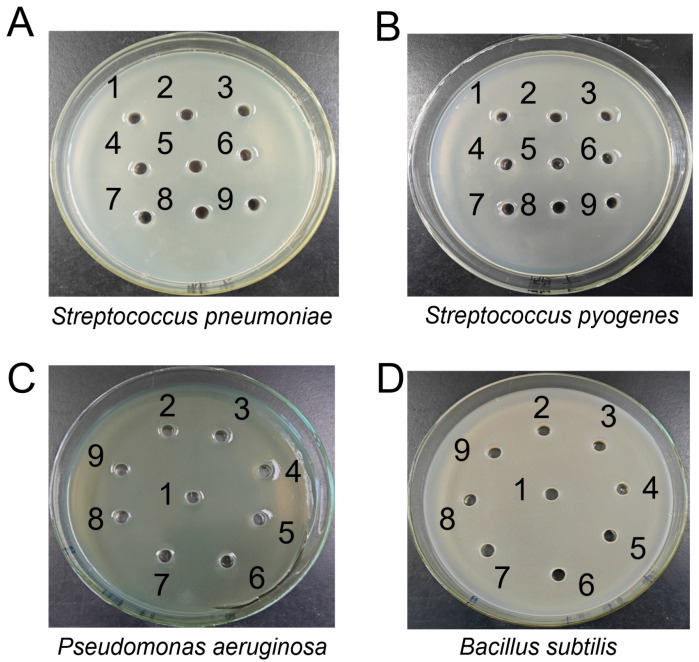
Bioassays of tryptophol against *Streptococcus pneumoniae* (**A**), *Streptococcus pyogenes* (**B**), *Pseudomonas aeruginosa* (**C**), and *Bacillus subtilis* (**D**). An amount of 50 μL of mixed solvent (methanol: water = 1:1) was added into hole 1 as a negative control. Different concentrations of tryptophol that serially diluted at a 1:1 ratio (7.81, 15.63, 31.25, 62.5, 125, 250, 500, 1000 μg/mL) were added into holes 2–9, respectively.

**Figure 4:**
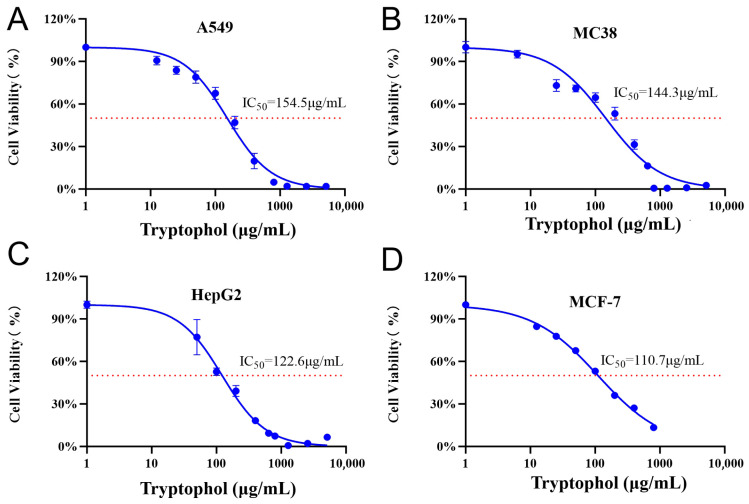
Cytotoxicity assays of tryptophol against A549 (**A**), MC38 (**B**), HepG2 (**C**), and MCF-7 (**D**) cells. A549: human lung cancer cells; MC38: mouse colorectal cancer cells; HepG2: human liver cancer cells; and MCF-7: human breast cancer cells. IC_50_ (50% inhibitory concentration) values are indicated in the figures. Error bars represent the standard deviations (SDs) of three independent experiments.

To investigate the biological activities of tryptophol against some pathogenic bacteria, bioassays were conducted using *Streptococcus pneumoniae* 010, *Streptococcus pyogenes* #2, *Pseudomonas aeruginosa* PA14, and *Bacillus subtilis* 1.1630. However, no obvious inhibition zones were observed, even at the tested maximum concentration of tryptophol (1000 μg/mL) (Figure 3A–D). Subsequently, the antitumor activities were detected, and 50% inhibitory concentrations (IC_50_) against A549, MC38, HepG2, MCF-7, and THP-1 were determined as described previously [19]. Tryptophol solutions dissolved in DMSO at various concentrations were incubated with pre-cultured A549, MC38, HepG2, MCF-7, and THP-1 cells to assess inhibitory effects. As a result, tryptophol exhibited obvious inhibitory activities against A549, MC38, HepG2, and MCF-7 to varying degrees, with IC_50_ values of 154.5 μg/mL, 144.3 μg/mL, 122.6 μg/mL, and 110.7 μg/mL, respectively (Figure 4A-D), whereas no obvious antitumor activity was detected against THP-1 cells (Figure S3). These findings revealed that tryptophol may exhibit potential for application in the future healthcare field.

### 3.4. Biosynthetic Pathway and Heterologous Expression of Tryptophol

The biosynthesis of tryptophol involves two main stages: the biosynthetic pathway and the utilization pathway of tryptophan. In the L-tryptophan biosynthetic pathway, glucose undergoes central carbon metabolism to generate phosphoenolpyruvate and erythrose-4-phosphate. These compounds then condense to form 3-deoxy-D-arabino-heptulosonate 7-phosphate, which is subsequently processed into shikimate and chorismic acid via the shikimate pathway. Chorismic acid then enters the L-tryptophan metabolic pathway for the final production of tryptophan. In the second stage, known as the Ehrlich pathway, tryptophan is converted to indole-3-pyruvic acid (IPyA) by aromatic amino acid transaminases (e.g., Aro8 and Aro9). IPyA is then decarboxylated by pyruvate decarboxylases (e.g., Aro10, Pdc1, Pdc5, Pdc6) to generate indole-3-acetaldehyde (IAAld). Finally, IAAld is reduced to tryptophol by multiple NAD(P)H-dependent dehydrogenases, including Adh1-5, Sfa1, and Aads [16,21].

**Figure 5:**
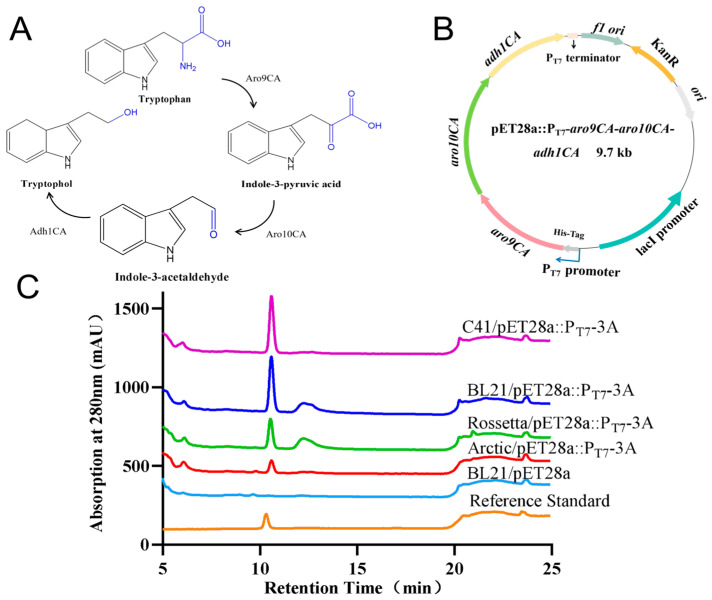
Biosynthetic pathway and heterologous expression of tryptophol. (**A**) Biosynthetic pathway of tryptophol from tryptophan; (**B**) codon optimization and construction of plasmid pET28a::P_T7_-*aro9CA-aro10CA-adh1CA*, abbreviated as pET28a::P_T7_-3A; (**C**) HPLC analyses of tryptophol produced by different *E. coli* strains. pET28a:P_T7_-3A was transformed into four expression hosts Arctic, Rossetta, BL21(DE3), and C41(DE3) to generate engineered strains Arctic/pET28a:P_T7_-3A, Rossetta/pET28a:P_T7_-3A, BL21/pET28a:P_T7_-3A, and C41/pET28a:P_T7_-3A, respectively.

By aligning the amino acid sequences of Aro9, Aro10, and Adh1 from the tryptophol biosynthetic pathway in *Saccharomyces cerevisiae* with their corresponding homologs from *S. longshengensis* and *C. albicans*, we discovered that silent tryptophol produced by *C. albicans* is induced by *S. longshengensis*, but the detailed mechanism remains unclear. Since tryptophol is a valuable compound, we expect to achieve its heterologous expression. Because the whole genome sequence data for *C. albicans* 2.4159 are not completely sequenced, we had to use *C. albicans* SC5314 as a reference strain to analyze the precise locations of genes coding the three key enzymes (Table 4) and to express them heterologously. Given that pathogenic *C. albicans* is unsuitable for strain improvement and industrial production, we selected *Escherichia coli* as the heterologous expression host due to its shorter reproduction cycle, simpler genetic manipulation, and less complex genetic background. We first selected and optimized the codon sequences of the key tryptophol biosynthetic genes *aro9CA* (C405560CA), *aro10CA* (CR06860CA), and *adh1CA* (C505050WA) and constructed the expression vector pET28a::P_T7_-*aro9CA*-*aro10CA*-*adh1CA* driven by the T7 promoter (abbreviated as pET28a::P_T7_-3A) (Figure 5A,B).

To investigate whether the tryptophol synthase gene can be expressed in *Escherichia coli*, and to determine the most suitable host strain for expression, the plasmid pET28a:P_T7_-3A was respectively introduced into the four *E. coli* strains—Arctic, Rosetta, BL21(DE3), and C41(DE3)—using BL21/pET28a as a control. HPLC analyses revealed that C41/pET28a:P_T7_-3A and BL21/pET28a:P_T7_-3A exhibited higher yields compared with the other strains (Figure 5C), implying that the biosynthetic genes involved in tryptophol production from *C. albicans* are successfully expressed in *E. coli* C41/pET28a:P_T7_-3A and BL21/pET28a:P_T7_-3A; the constructed genetic expression system can be employed for further strain improvement and yield enhancement. The resulting knowledge was used not only to understand how this complex molecule is made, but also to engineer the producing organism to make potentially improved derivatives.

## 4. Discussion

Tryptophol exhibits potential for application and commercial value across multiple fields. Indole derivatives exhibit inhibitory efficacy against drug-resistant pathogens and cancer cells, including inhibitory effects on bacteria, fungi, and viruses, as well as antioxidant, anti-inflammatory, and antitumor properties. In pharmaceutical applications, tryptophol analogs serve as common intermediates for numerous important medicinal compounds. Examples include the triptan family of anti-migraine drugs (such as sumatriptan, rizitriptan, and zolmitriptan) and etodolac with a pyranoindole architecture that exhibits anti-inflammatory and analgesic effects [15]. Studies by Schirmer et al. revealed tryptophol’s ability to inhibit cytokine production (TNFα and IFNγ) induced by pathogens such as *C. albicans* [22], while Elleuch et al. confirmed its antibacterial effects against *Fusarium* sp. and *S. enterica* ATCC 43972 [23]. Meanwhile, no inhibition zone was observed in the bioassays of antimicrobial activities of tryptophol against *Streptococcus pneumoniae*, *Streptococcus pyogenes*, *Pseudomonas aeruginosa*, and *Bacillus subtilis*. We speculated that the antibacterial activities of tryptophol may be associated with their mechanism of action against bacteria, bacterial resistance, and the working concentration of tryptophol.

This study further demonstrates tryptophol’s inhibitory effects on HepG2, A549, MCF7 and MC38. These findings indicate the potential efficacy of tryptophol against these cancer cells. Tryptophol shows some advantages such as low toxicity and ease of structural modification. It is anticipated that, in the future, more tryptophol analogs wound have increased opportunities to enter clinical treatment and benefit human health.

A co-culture system was used to explore novel natural products, and we accidentally found that tryptophol biosynthesis was activated during this process. Tryptophol is an important secondary metabolite and signaling molecule that can be produced by *C. albicans* and *Saccharomyces cerevisiae*. Its biosynthesis may be blocked or significantly reduced under conditions of nutrient deprivation, changes in culture conditions, or modulation of negative regulators in the pathway, and the underlying mechanisms are usually complex. Since MS solid medium is commonly used for the cultivation of *Streptomyces* but not for *C. albicans*, the barren living environment led to the cessation of the production of tryptophol in the monoculture of *C. albicans*. Co-culture of the two strains induced the emergence of tryptophol, implying that the complementarity of relevant activating factors between cross-kingdom microorganisms is significant, but its mechanism of action is intricate. We further hypothesized that *S. longshengensis* itself, in response to environmental stress, induces the production of tryptophol, which may function as a quorum sensing molecule to modulate fungal morphogenesis as well as biofilm formation [24,25,26]. In this study, tryptophol was activated through the co-cultivation of cross-kingdom microorganisms between *S. longshengensis* and *C. albicans* on MS solid medium, resulting in higher yields compared with liquid cultures and more closely mimicking natural soil or habitat conditions. The mechanisms by which microbial interactions trigger silent activation of previously suppressed metabolic pathways are diverse and may include signal molecule exchange, stress responses, precursor or intermediate supply, and activation of regulatory pipelines. *S. longshengensis* may provide certain precursor substances for tryptophol biosynthesis or respond to some kind of pressure signal; however, the specific mechanisms remain unknown. Microbial co-cultivation can serve as an alternative to traditional monoculture methods because it promotes the production of extra metabolites that individual microbial strains are unable to generate.

Here, tryptophol biosynthetic genes from *C. albicans* were successfully highly expressed in *E. coli* C41, but the yield of tryptophol produced by microbial chassis cells is still low. Guo et al. reported that the recombinant *E. coli* strain DG111, which was used for the co-expression of YjgB, KDC, and ARO8, produced 653 ± 28.99 mg/L of tryptophol [17]. There remains substantial potential for yield enhancement, such as through CRISPR gene editing, to further optimize tryptophol biosynthetic pathways, along with process optimizations including substrate feeding, pH regulation, and increased solubility.

## 5. Conclusions

This study reports bioactive natural compounds produced by co-cultivating cross-kingdom *S. longshengensis* and *C. albicans*. Using MS and NMR spectroscopy, a valuable compound tryptophol was identified, showing potential antitumor effects (IC_50_ values ranging from 110.7 to 154.5 μg/mL against A549, MC38, HepG2, and MCF-7 cell lines). Tryptophol can be produced through heterologous expression in *E. coli* C41(DE3), addressing the limitations of chemical synthesis and providing a basis for its sustainable industrial production.

## Figures and Tables

**Table 1:** Linear elution gradient of acetonitrile and water in general HPLC analysis.

Time (min)	0	5	35	40	40.10	45
Solvent A: water (%)	95	95	5	5	95	95
Solvent B: acetonitrile (%)	5	5	95	95	5	5

**Table 2:** Linear elution gradient of acetonitrile and water used for large-scale preparation in HPLC analysis.

Number of Separations	Time (min)	0	5	40	40.10	45	45.10	50
The first separation conditions	Solvent A: water (%)	80	80	20	5	5	80	80
	Solvent B: acetonitrile (%)	20	20	80	95	95	20	20
The second separation conditions	Solvent A: water (%)	80	80	79	5	5	80	80
	Solvent B: acetonitrile (%)	20	20	21	95	95	20	20

**Table 3:** Linear elution gradient of acetonitrile and formic acid solution in HPLC analysis.

Time (min)	0.00	0.50	11.50	12.50	13.50	15.00
0.1% (*v*/*v*) formic acid solution (%)	90	90	75	0	0	90
acetonitrile (%)	10	10	25	100	100	10

**Table 4:** Homologous alignment of key enzymes in the tryptophol biosynthetic pathway of *C. albicans*.

Catalytic Steps	Protein	Position	Identity	Descriptions
Transamination	Aro8CA (C200340CA)	Chromosome 2	52.52%	bifunctional 2-aminoadipate transaminase
	Aro9CA (C405560CA)	Chromosome 4	36.33%	aromatic-amino-acid:2-oxoglutarate transaminase
Decarboxylation	Aro10CA (CR06860CA)	Chromosome R	35.52%	phenylpyruvate decarboxylase
Dehydrogenation	Adh1CA (C505050WA)	Chromosome 5	73.78%	alcohol dehydrogenase
	Adh2CA (C108330CA)	Chromosome 1	76.72%	alcohol dehydrogenase
	Adh3CA (C204470WA)	Chromosome 2	23.96%	alcohol dehydrogenase

## Data Availability

The original contributions presented in this study are included in the article. Further inquiries can be directed to the corresponding authors.

## Supplementary Materials

The following supporting information can be downloaded at: https://www.mdpi.com/article/10.3390/microorganisms14040798/s1, Figure S1: HPLC analysis of crude extracts from two biological replicates of the co-culture system of *Streptomyces longshengensis* 4.1101 and *Candida albicans* 2.4159; Figure S2: Standard curves of tryptophol; Figure S3: Cytotoxicity assay of tryptophol against THP-1 cells.

## Abbreviations

The following abbreviations are used in this manuscript:

HPLC	High-performance liquid chromatograph
MS	Mass spectrum
NMR	Nuclear magnetic resonance
SMs	Secondary metabolites
A549	Human lung cancer cells
MC38	Mouse colorectal cancer cells
HepG2	Human liver cancer cells
MCF-7	Human breast cancer cells
THP-1	Human acute monocytic leukemia cells
HRESI-MS	High-resolution electrospray ionization mass spectrometry
IC_50_	50% inhibitory concentrations
