# Supplementary material for: Co-Cultivation of Cross-Kingdom Microorganisms Effectively Triggers the Production of Tryptophol and Its Heterologous Expression in E. coli
Source: Microorganisms. 2026 Apr 1;14(4):798. doi: 10.3390/microorganisms14040798 (PMC13118851; doi:10.3390/microorganisms14040798)
Supplement: Supplementary file 1 [file microorganisms-14-00798-s001.zip › microorganisms-4199678-supplementary.pdf]

Supplementary information for

Article

Co-cultivation of cross-kingdom microorganisms effectively triggers the production of tryptophol and its heterologous expression in *E. coli*

Yue Li [1†,*], Xiulei Xia [2†], Jinwei Ren [3], Huarong Tan [2], Jine Li [2*]

[1] Beijing Institute of Dental Research, Beijing Stomatological Hospital, Capital Medical University, Beijing 100070, China; liyue200906@163.com(Y.L.);

[2] State Key Laboratory of Microbial Diversity and Innovative Utilization, Institute of Microbiology, Chinese Academy of Sciences, Beijing 100101, China; 15668393609@163.com (X.X.); tanhr@im.ac.cn (H.T.); lijine@im.ac.cn (J.L.)

[3] State Key Laboratory of Mycology, Institute of Microbiology, Chinese Academy of Sciences, Beijing, 100101, China; renjw@im.ac.cn (J.R.).

* Correspondence: liyue200906@163.com(Y.L.); lijine@im.ac.cn (J.L).;

† These authors contributed equally to this work.

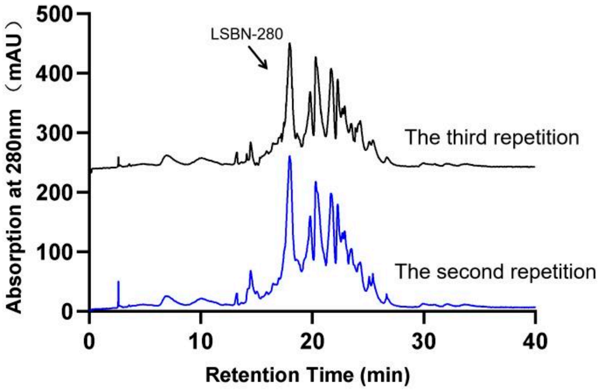

Figure S1 HPLC analysis of crude extracts from two biological replicates of the co-culture system of *Streptomyces longshengensis* 4.1101 and *Candida albicans* 2.4159. Ultraviolet (UV) absorption was monitored at 280 nm. LSBN-280 represents the differential metabolite peak.

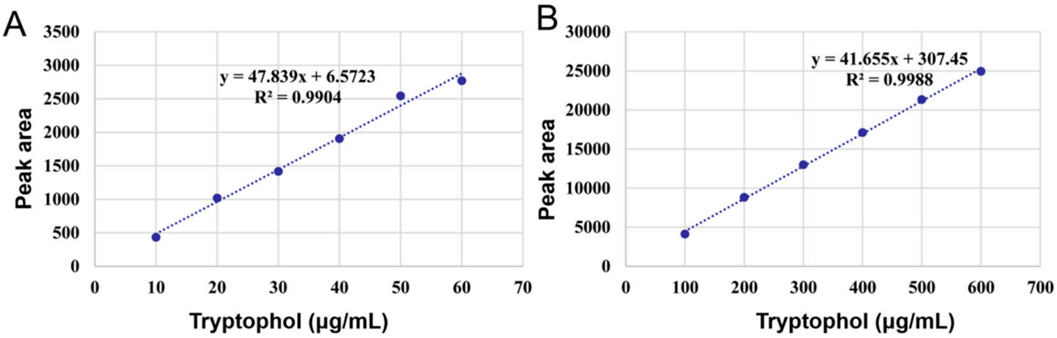

Figure S2 Standard curves of tryptophol. (A) Standard curve for low-concentration tryptophol, with a linear range of 0-70 μg/mL. (B) Standard curve for high-concentration tryptophol, with a linear range of 100-700 μg/mL.

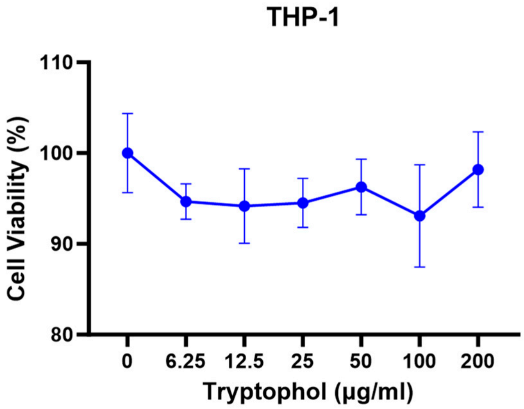

Figure S3 Cytotoxicity assay of tryptophol against THP-1 cells. THP-1: the human acute monocytic leukemia cell line. Error bars represent the standard deviations (SD) of three independent experiments.
